# Concerns about the dengue epidemic

**DOI:** 10.31744/einstein_journal/2025CE1632

**Published:** 2025-05-30

**Authors:** Mariana Hayashi Garcia, Paulo Bandiera-Paiva, Luciano Rodrigo Lopes

**Affiliations:** 1 Universidade Federal de São Paulo Escola Paulista de Medicina Department of Health Informatics São Paulo SP Brazil Department of Health Informatics, Escola Paulista de Medicina, Universidade Federal de São Paulo, São Paulo, SP, Brazil.

Dear Editor,

For several decades, the dengue epidemic has been a concern for public health authorities. The dengue virus (DENV) is endemic to tropical and subtropical regions, and its annual incidence has increased significantly over the years.^(1)^ Since it is transmitted by an arthropod vector (the *Aedes aegypti* mosquito), it is classified as an arbovirus, along with the yellow fever, chikungunya, and Zika viruses. There are four serotypes of the virus (DENV-1 to DENV-4), all of which can infect humans.^(2)^

**Figure 1:**
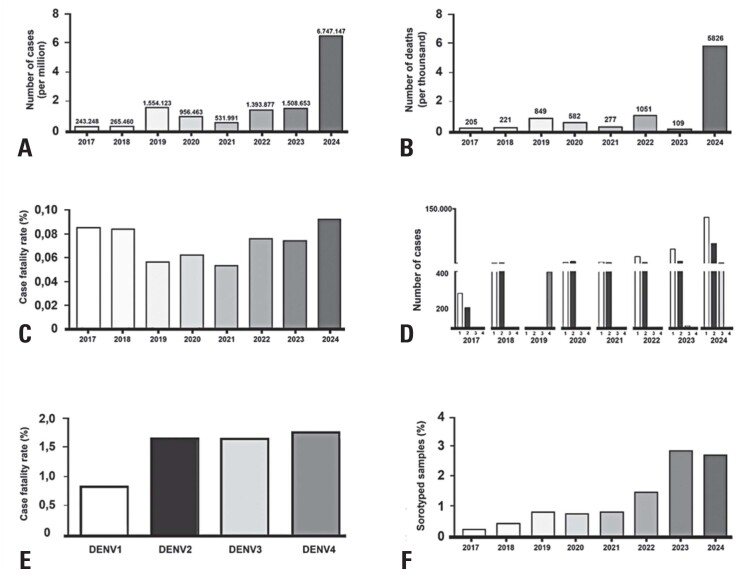
(A) number of dengue cases and (B) deaths, and (C) case fatality rate from 2017 to 2024; (D) number of cases by serotype (DENV-1 to DENV-4) from 2017 to 2024; (E) case fatality rate according to the serotype; (F) percentage of serotyped samples per year. The graphs were generated using GraphPad Prism V.5 software based on data extracted from the DATASUS website

In 2024, DENV exhibited high transmissibility, becoming a major concern for Brazilian society and public health.^(3)^ Based on official Brazilian government data available on the DATASUS website, the number of dengue cases and deaths in Brazil substantially exceeded those in previous years (Figure 1A and B). The case fatality rate of the dengue virus, calculated as the number of deaths divided by the number of cases, was notably higher in 2024 (Figure 1C). DENV-1 was the predominant serotype, although DENV-2 was also present in markedly high numbers (Figure 1D). Additionally, there has been an increase in the number of DENV-3 cases. Notably, both DENV-2 and DENV-3 showed higher fatality rates than DENV-1 (Figure 1E). Case and death quantification were performed using the codes A90 (Dengue Fever) and A91 (Dengue Hemorrhagic Fever) from the ICD-10 system (International Classification of Diseases, 10th Revision) on the DATASUS website.

The scenario of a higher prevalence of a serotype with a higher fatality rate by 2025 is alarming. Considering that dengue infections placed high pressure on public healthcare resources in 2024, at times compromising its functionality, the possible predominance of more lethal serotypes throughout 2025 is a cause for concern. Thus, if the dengue outbreak in 2025 reaches levels as high as those of the previous year and is predominantly more lethal serotypes, disease containment strategies orchestrated by the Brazilian Ministry of Health will need to be even more effective, such as mass distribution of the dengue vaccine, vector control, and rapid diagnosis.

It is essential to emphasize the need for public policies to increase the serotyping rate of dengue cases. In 2024, less than 3% of the samples were serotyped (Figure 1F). This limitation can affect the accuracy of fatality rate estimation among serotypes, considering that a low serotyping rate may not be representative. Monitoring circulating serotypes provides a better understanding of the epidemiological scenario, allowing for the prediction of more severe outbreaks and guiding control and vaccination strategies.
